# Ultrasonographic appearance of the ileocecocolic junction in cats with salmonellosis

**DOI:** 10.3389/fvets.2025.1588445

**Published:** 2025-05-02

**Authors:** Magdalena Brzozowska, Martin Rapp, Simon Vermeire, Daniel Klich, Ewa Stanczyk

**Affiliations:** ^1^Evidensia Small Animal Referral Hospital, Strömsholm, Sweden; ^2^Department of Animal Genetics and Conservation, University of Life Sciences, Warsaw, Poland; ^3^Vet Oracle, Norfolk, United Kingdom

**Keywords:** feline, ultrasound, ileocecocolic junction, salmonellosis, diagnostic imaging

## Abstract

Salmonellosis is a bacterial infection caused by Salmonella spp. When it affects the gastrointestinal tract of cats, it has a predilection for the ileocecocolic junction. Abdominal ultrasound is a valuable tool in diagnosing gastrointestinal diseases. This retrospective, single-center case series study aimed to describe the ultrasonographic abnormalities of the ileocecocolic junction in feline patients with confirmed Salmonella infection. One hundred cats presenting with gastrointestinal symptoms were tested for Salmonella between 2019 and 2022; 70 tested positive, and 30 tested negative. All of them underwent ultrasonographic examination. The positive group showed a significantly thickened wall of the ileum (*p* < 0.001, range: 1.9–5.7 mm, mean = 3.25 mm, SD = 0.66), caecum (*p* < 0.001, range: 2.1–3.9 mm, mean = 2.87 mm, SD = 0.49), and ascending colon (*p* < 0.05, range: 1–3.1 mm, mean = 1.47 mm, SD = 0.55) in comparison to the negative group. In the positive group, the ileocecal lymph nodes were significantly larger in diameter (*p* < 0.001, range: 2.7–7.8 mm, mean = 4.9 mm, SD = 1.15). All Salmonella-positive cats had focal hyperechoic mesenteric fat, and 23% (16/70) had focal peritoneal effusion at the ileocecocolic junction. The combination of the thickening of the intestinal wall at the ileocecocolic junction, focal hyperechoic mesenteric fat, peritoneal effusion and regional lymphadenopathy may indicate Salmonellosis in cats with compatible clinical signs. Including this diagnosis may prompt further testing, leading to early recognition and effective treatment, resulting in better patient outcomes. Diagnosing this disease is also important as Salmonellosis carries a zoonotic risk, with the potential transmission between pets and humans.

## 1 Introduction

Salmonella infections are a leading cause of human foodborne diseases in the world (1), with 1.3 billion people affected annually (2). Even though the majority of Salmonella serovars are pathologic to humans, animals can be asymptomatic carriers that contaminate the environment by shedding bacteria intermittently and as a result cause Salmonellosis through pet-to-human transmission (2). Healthy adult cats show high immunological resistance to bacteria, which makes Salmonellosis uncommon in this species (3, 4). Infection is often favored by host immunosuppression, age, hospitalization, multicat environment, chemotherapy, exogenous glucocorticoid therapy or gestational status (4, 5). The main routes of infection include fecal-oral transmission, ingestion of contaminated food, raw-meat diet, ocular transmission, possibly aerosolization and transplacental transfer (4, 6). Additional sources of infection for outdoor cats include scavenging and hunting rodents and birds, exposure to reptiles and environmental contamination (7). Four scenarios may follow infection: transient asymptomatic infection, acute gastroenteritis, carrier state, or bacteremia. Clinical signs vary with the number of indigested organism and their virulence, as well as the immune status of the host (8). Cats are primarily infected subclinically, but gastrointestinal disease can occur and is initially associated with fever, malaise and anorexia, followed by vomiting, abdominal pain and diarrhea (9). Less commonly abortion, stillbirth, meningoencephalitis, respiratory distress, conjunctivitis and myocarditis with endocarditis can occur in cats with Salmonellosis (5, 10, 11). Finally, some infected individuals manifest no or mild transient clinical symptoms and can become long-term chronic carriers (8).

The current literature has been focusing on epidemiologic or clinical aspects of the infection (4, 5, 7, 12, 13). Described gross pathological lesions in cats were thickening of the wall of the terminal ileum, abnormal content in the ileum and colon, and reddened and enlarged mesenteric lymph nodes (14). Histologically the colonic mucosa had cryptitis and leucocyte infiltration (14). Typhlitis and jejunitis characterized by lymphoplasmacytic and neutrophilic inflammation are also documented (8).

A descriptive study of ultrasonographic changes in feline patients with Salmonellosis is however lacking. The aim of this study is to describe the ultrasonographic abnormalities of the ileocecocolic junction in feline patients with confirmed Salmonellosis. We hypothesized that cats suffering from acute gastrointestinal disease due to Salmonella infection would show ultrasonographically detectable alterations near the ileocecocolic junction.

## 2 Materials and methods

In this retrospective, descriptive study, the database of the Evidensia Small Animal Referral Hospital in Strömsholm, Sweden was searched for cats tested for Salmonella infection between January 2019 and October 2022. Cats were included in the study if they presented with clinical symptoms indicative of acute gastrointestinal disease, underwent abdominal ultrasonographic examination documenting the ileocecocolic junction, and were tested for Salmonella infection.

Medical records were searched, and information regarding the patient’s signalment, breed, age, sex, outdoor vs. indoor vs. indoor/outdoor style of living, clinical presentation, results for fecal Salmonella sampling and ultrasonographic examination were recorded by veterinary diagnostic imaging resident.

All cats were fasted prior to the ultrasound examination and positioned in dorsal recumbency. The fur was clipped, alcohol, and ultrasonographic gel were applied to the skin of the abdomen. During the scan, the majority of the cats were conscious. All the studies were performed on the same machine (Logiq E9, GE Healthcare, Wisconsin, US) using linear 9-11 MHz and 6-10 MHz microconvex probes by various experienced small animal ultrasonographers with more than 5 years of experience.

Static images and cine loops were reviewed in PACS viewing software (IDS7, Sectra AB, Linköping, Sweden). The following parameters were recorded: the thickness (mm) of the ileal, cecal and ascending colonic walls, measured on sagittal plane images, from the serosal to mucosal surface, wall layering (reduced or preserved), echogenicity of the mesenteric fat (normal or hyperechoic compared to normal mesenteric fat), presence of peritoneal effusion (present or absent) and the maximum dimension of ileocecal lymph nodes (mm).

The cats were divided into positive and negative groups based on the results of Salmonella bacterial fecal testing. The samples were analyzed by a referral laboratory (The Swedish Veterinary Agency, SVA, Uppsala, Sweden) with bacterial culture ISO 6579:2017–1.

Four variables; wall thickness of ileum, caecum and ascending colon, and lymph node size, were tested for normality using the Shapiro–Wilk test. Variables that were normally distributed were tested with the student’s *T*-test, and others were tested with the Mann–Whitney *U* test. Values of the variables for positive and negative Salmonella groups were compared. A *p*-value < 0.05 was considered statistically significant. Statistical analysis was performed in SPSS software v. 28 (Armonk, New York) by a statistician.

## 3 Results

One hundred cats met the inclusion criteria. Seventy tested positive, while thirty tested negative for Salmonella infection. All patients presented with symptoms of inappetence, vomiting, diarrhea, dehydration, abdominal pain and fever. The salmonella-positive group included European shorthair cats (58/70), Siberian cats (4/70), Ragdolls (3/70), Norwegian forest cats (2/70), Maine coon (1/70), LaPerm (1/70) and Neva masquerade (1/70). The median age was 2.4 years (6 months-13 years). Sixty-nine cats were indoor/outdoor cats, while one was strict indoor. The Salmonella-negative group included European shorthair cats (27/30), Devon rex (1/30), Siberian (1/30) and a Norwegian forest cat (1/30). The median age was 3.8 years (1–14 years). All individuals were indoor/outdoor cats.

The positive group showed a significantly thicker total wall of the ileum (*p* < 0.001, range: 1.9–5.7 mm, mean = 3.25 mm, SD = 0.66), caecum (*p* < 0.001, range: 2.1–3.9 mm, mean = 2.87 mm, SD = 0.49), and ascending colon (*p* < 0.05, range: 1–3.1 mm, mean = 1.47 mm, SD = 0.55) in comparison to the negative group [total ileal thickness (range: 1.4-4 mm, mean = 2.3 mm, SD = 0.56), cecal thickness (range: 1.7–3.2 mm, mean = 2.26 mm, SD = 0.41) and thickness of the ascending colon (range: 1–2.4 mm, mean = 1.18 mm, SD = 0.36)].

**Figure 1:**
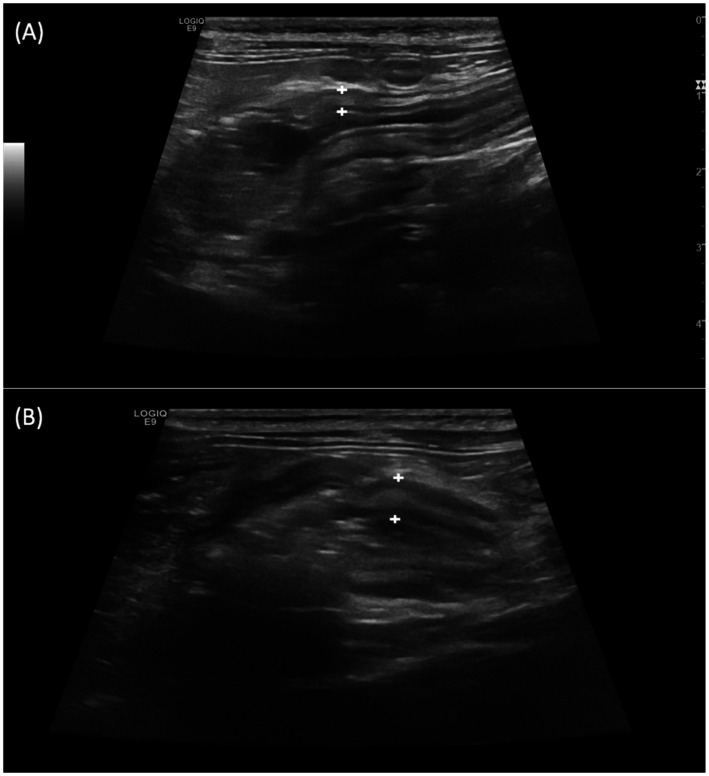
Longitudinal scan over the ileocecocolic junction from two different patients **(A,B)** from Salmonella-positive group documenting thickened ileal wall (between the calipers).

**Figure 2:**
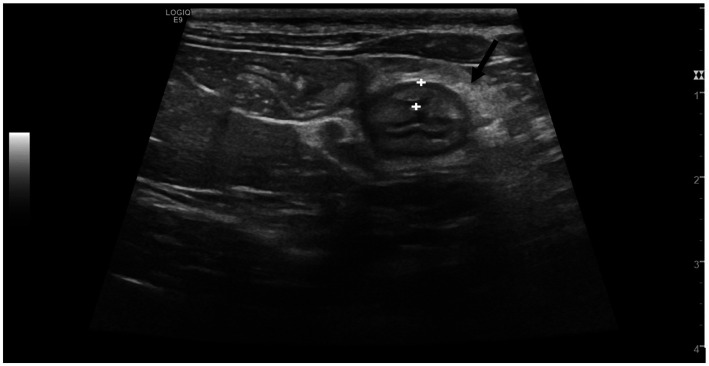
Transverse scan of the ileum of a cat from Salmonella-positive group, documenting the thickened ileal wall (between the calipers). The black arrow points at hyperechoic mesenteric fat.

**Figure 3:**
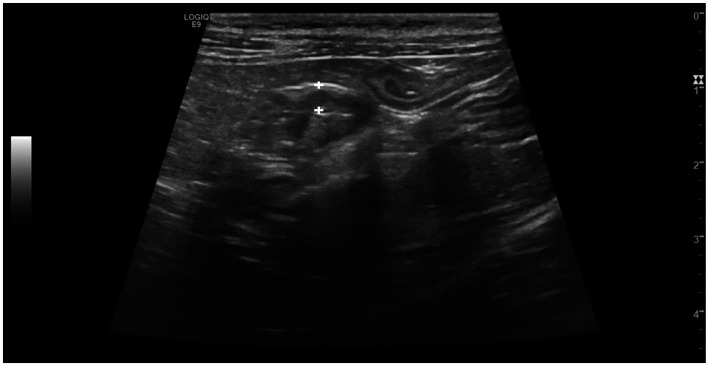
Longitudinal scan of the caecum of a cat from Salmonella-positive group, documenting the thickening of the caecal wall (between the calipers).

Despite the thickening of the intestinal wall, the wall layering was preserved. The ileal and colonic walls showed segmental, circumferential symmetrical thickening (Figures 1, 2). The cecal wall thickening was circumferential and symmetrical (Figure 3).

**Figure 4:**
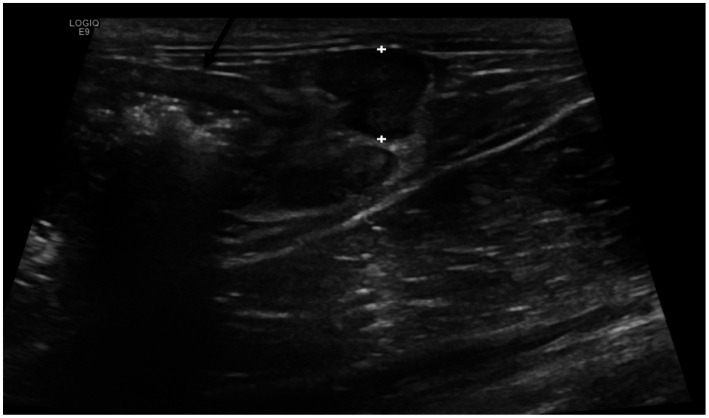
Enlarged and hypoechoic ileocecal lymph nodes (between calipers) located by the thickened ascending colon (black arrow) in a cat from Salmonella-positive group.

In the positive group, the ileocecal lymph nodes were significantly larger (*p* < 0.001, range: 2.7–7.8 mm, mean = 4.9 mm, SD = 1.15, Figure 4), in comparison to the negative group (range: 2-6 mm, mean = 3.3 mm, SD = 1.03).

In all Salmonella-positive cats, mesenteric fat at the ileocecocolic junction showed increased echogenicity compared to the normal fat, and 23% of patients (16/70) had a focal peritoneal effusion.

## 4 Discussion

To the author’s knowledge, this is the first clinical study assessing ultrasonographic changes of the ileocecocolic junction in cats with confirmed Salmonella infection. The results indicate that affected cats exhibit a significantly increased thickness of the wall of the ileum, caecum and ascending colon, along with lymphadenopathy of the ileocecal lymph nodes, hyperechoic mesenteric fat and, in some cases, focal peritoneal effusion. The patients showed no ultrasonographic changes in the remainder of the gastrointestinal tract.

Abdominal ultrasound is routinely used for imaging of the feline gastrointestinal tract. The intestinal wall thickness, and preservation or loss of the wall layering can be easily assessed during the scan. Moreover, ultrasound helps visualize luminal content, detect intestinal dilation and assess peristalsis. The structures like peritoneum and regional lymph nodes can be evaluated simultaneously (15).

Ileocecocolic pathologies are a relatively frequent finding in cats with gastrointestinal diseases (16). The infectious causes of abnormal ileocecocolic junction include feline infectious peritonitis, parasites such as Trichuris species, Ancylostoma species, Tritrichomonas foetus, Cryptosporidium parvum, Giardia duodenalis, Isospora species, Toxoplasma gondii and, much less frequently, bacterial enterocolitis (Campylobacter species, Escherichia coli, Salmonella species, Yersinia species, Clostridium species) (17). Other less common causes include inflammatory diseases, intussusception of the caecum into the colon, stricture, neoplasia and fecaliths (17–20). Alimentary lymphoma is the most common intestinal neoplasm in cats (55%), followed by adenocarcinoma (32%), and mast cell tumor (4%) (21). In cats, highly predictive for focal intestinal neoplasia is wall thickening and loss of layering (22). Solitary, hypoechoic intestinal masses with transmural loss of layering commonly represent intermediate- and high-grade lymphoma (21, 23). In the case of adenocarcinoma, the thickening of the intestinal wall is often segmental, and circumferential, with transmural loss of layering and lymphadenopathy (22). Our study population showed preserved wall layering which would be a helpful feature to differentiate from neoplasia. Similar to Salmonellosis the adenocarcinoma most frequently occurs at the ileocecocolic junction, followed by the jejunum and ileum (22). In cases of lymphoma the most common site is the small intestine, but the preferred segment depends on the molecular clonality (24, 25). High-grade alimentary lymphoma is B- or T-cell origin. T-cell lymphoma is most common in the ileum and jejunum (24, 25). B-cell lymphoma affects the stomach, caecum and colon (24, 25). Low-grade lymphoma is usually associated with less aggressive ultrasound findings, such as diffuse wall thickening with preserved layering and relative thickening of the muscular layer (23). It can be mistaken with inflammatory bowel disease, eosinophilic or lymphocytic enteritis which also cause thickening of the muscular layer with preserved layering (23, 26). In older cats, thickening of the muscular layer is more likely to indicate lymphoma rather than inflammatory bowel disease (23). The mast cell tumor is the third most common primary intestinal tumor in cats. The duodenum, jejunum, ileocecocolic junction and colon can be affected with ultrasonographic alterations mainly affecting the muscular layer (27).

In human ultrasonographic findings related to Salmonella include splenomegaly, enlarged mesenteric lymph nodes, intestinal wall thickening, acalculus cholecystitis and hepatomegaly, but thickened terminal ileum or proximal colon with mesenteric lymphadenopathy are considered most typical (28). The intestinal wall thickening is due to oedema of mucosa and submucosa with preserved layering (29). On computed tomography in human, the ileal wall thickening is symmetric, homogenous and can be focal or diffuse (28). Salmonella-induced terminal ileitis can mimic Crohn’s disease (30). It is interesting that the terminal ileum, colon or caecum are the predilection sites for both cats and humans which is most likely due to the pathogenesis of the disease. Salmonella preferentially attaches to and invade ileal villi (8). The intestinal M-cells within the lymphoid-follicle associated epithelium are the primary site of the invasion (31).

During infection bacteria enter its host and penetrates the Peyer’s patches using specialized antigen sampling M-cells of the mucosal immune system (32–34). After penetration bacteria invade the mucosa by crossing the epithelial barrier, translocate to the intestinal lymphoid follicles and the draining mesenteric lymph nodes and some pass to the liver and spleen (32, 35). In human the most common sites of infection are Peyer patches of terminal ileum, gallbladder, liver, spleen, caecum and bone marrow (32, 35). In cats the accumulation of Peyer patches is described in the submucosa of the distal jejunum and ileum, which can be observed via ultrasound (36). In addition the feline caecum is composed of numerous lymphoid follicles (16). This could aid in understanding the preferred and repeatable predilection site of the ileocecocolic junction for Salmonella in our study group.

Disorders of the ileocecocolic junction can be found in all age categories (17, 18) which is also seen in our study population. The European shorthair was dominant in our study population, probably because it is the most common breed presented for consultation, thus the high number reflects breed popularity and availability rather than susceptibility to infection. What is interesting in our study is that only one cat positive for Salmonella infection was strictly indoors, while the other ones were both outdoor and indoor. It suggests that indoor/outdoor lifestyle increases the risk of infection, possibly through scavenging or hunting birds and rodents. However, the definitive source of infection remains unknow in our study. There are described seasonal Salmonella outbreaks in 1999, 2009–2016 in Sweden (37, 38). The authors of these papers emphasized the outdoor lifestyle of cats in Sweden with close proximity to nature and birds. Infected birds can spread the disease at garden feeders. Moreover, birds dying from septicaemia are easy prey for house cats, which would get infected with high doses of Salmonella (37, 38). One cat from our study population was strictly indoors; possible infection routes included a raw meat-based diet, contaminated items brought to the house or contact with a subclinically infected animal.

The combination of an acute onset of clinical signs, including inappetence, diarrhea, dehydratation, abdominal pain, and fever, along with a fecal culture positive for Salmonella, provides a relatively confident diagnosis (39, 40). Consequently, tests for other diseases that might cause an acute onset of enteropathy were not performed on patients in the Salmonella-positive group. Also, patients were not testes for comorbidities that could result in ultrasonographic changes at the ileocecocolic junction. We cannot completely rule out that other pathologies, such as inflammatory or infectious enteropathies, could account for or contribute to the ultrasonographic changes observed in the Salmonella-positive group, especially given that most of the patients in this group were outdoor cats, which have a higher risk of contracting infectious diseases compared to indoor cats. The detection of Salmonella in the fecal sample does not imply that the patient suffers from Salmonellosis, as carrier animal can intermittently shed the bacteria (39). It cannot be entirely excluded that some of patients in the Salmonella-positive group could be carriers. However, the combination of clinical symptoms and the presence of Salmonella in the fecal sample most likely represents a patient in the acute phase of a disease, rather than a carrier animal suffering from other acute inflammatory/infectious gastrointestinal disease.

This study has several limitations. It is lacking testing for other infectious diseases and possible comorbidities and excluding potential Salmonella carriers. It is retrospective in nature, and no histological samples were obtained. There was no standardized timeframe for conducting an ultrasound after the onset of clinical symptoms. Follow-up ultrasound studies were not performed, which prevents establishing a correlation between the resolution of clinical symptoms and ultrasonographic abnormalities. As a result, there is no specific timeline indicating how long abnormalities persist on ultrasound. The absence of follow-up studies stems from the limited interest shown by the owners when the patient responded well to treatment and returned to normal. Further studies that incorporate the histopathology could be beneficial.

## 5 Conclusion

Salmonella is rare in cats but can be found in the feline population, particularly among hunting outdoor cats, most likely due to close contact with nature and birds. Although not specific, and overlapping with other diseases, the combination of thickening of the ileal-, cecal-, and ascending colonic walls, regional lymphadenopathy, focal hyperechoic mesenteric fat and peritoneal effusion, could indicate Salmonellosis, which should be considered as differential diagnosis in cats with gastrointestinal disease.

## Data Availability

The original contributions presented in the study are included in the article/supplementary material, further inquiries can be directed to the corresponding author.
